# Mapping the Paths from Styles of Anger Experience and Expression to Obsessive–Compulsive Symptoms: The Moderating Roles of Family Cohesion and Adaptability

**DOI:** 10.3389/fpsyg.2017.00671

**Published:** 2017-05-02

**Authors:** Liang Liu, Cuilian Liu, Xudong Zhao

**Affiliations:** ^1^Department of Clinical Psychology, Shanghai Pudong New Area Mental Health CenterShanghai, China; ^2^Center of Psychological Counseling, Tongji UniversityShanghai, China; ^3^Department of Clinical Psychology, Shanghai East Hospital, School of Medicine, Tongji UniversityShanghai, China

**Keywords:** obsessive–compulsive disorder, anger proneness, anger expression, family cohesion, family adaptability, moderation effect, gender

## Abstract

Previous research has shown strong connections of anger experience and expression with obsessive–compulsive (OC) symptoms. Additionally, studies have demonstrated links between family environment variables and obsessive–compulsive disorder (OCD). Our study aims to integrate the perspectives from these two literatures by exploring the moderating roles of family cohesion and family adaptability in the relationship between anger proneness and suppression and OCD symptoms. A total of 2008 college students were recruited from a comprehensive university in Shanghai, China between February and May 2016. The subjects completed self-report inventories, including the Symptom Check List-90, State-Trait Anger Expression Inventory 2 (Chinese version), and Family Adaptability and Cohesion Scale, second edition (Chinese Version). Controlling for age, one-child family status, ethnicity, family income, current depression, and anxiety, our analyses showed that the association between anger proneness and OC symptoms was moderated by family cohesion among men and that family adaptability moderated the connection between anger suppression and OC complaints among women. The findings imply that a more cohesive and empathic family environment may protect male students with high levels of anger proneness from developing OC behaviors or thoughts. The results suggest that for female subjects who are accustomed to suppressing angry feelings, flexible family coping strategies and communication atmospheres would reduce their vulnerability to OC symptoms. The findings are somewhat consistent with those of previous studies on psychotherapy outcomes that showed that OCD patients benefitted from psychotherapeutic interventions that cultivated the clients’ family cohesion and adaptability.

## Introduction

Obsessive–compulsive disorder (OCD) involves recurrent, unwanted and distressing thoughts (obsessions) and behaviors intended to relieve obsessional distress (compulsive rituals) (Whiteside and Abramowitz, 2004). The identification of the variables that contribute to the development and maintenance of obsessive–compulsive (OC) symptoms and the pathways from those risk factors to OC symptoms can inform the design of improved treatment strategies for individuals with OC thoughts or ritualistic behaviors.

Previous studies have shown that anger plays an important role in the development of OCD. Individuals with this disorder were more prone to experience anger and internalize their hostility than to express it outwardly (Whiteside and Abramowitz, 2005; Piacentino et al., 2016). However, this result does not suggest that all individuals with high levels of trait anger and anger suppression experience similar levels of OC pathologies. This heterogeneity implies that factors may moderate the associations between anger experience and OCD and may protect individuals with high levels of anger proneness and suppression from the disturbance of obsessions and compulsive rituals. Previous research has shown that family dynamics, including family support and flexible coping strategies, are empirically associated with OCD (Pens et al., 2012; Peris and Piacentini, 2013; Murphy and Flessner, 2015). The present study examines the hypothesis that the variances in family cohesion and family adaptability affect the strength of the links between trait anger and anger suppression and OC symptoms. In other words, among individuals reporting higher family cohesion or flexibility scores, anger proneness and suppression might be less significantly associated with OC symptoms and vice versa.

### Anger Proneness, Anger Expression, and OC Symptoms

Previous research conducted by Spielberger et al. (1985) and other researchers (Liu et al., 2011) demonstrated the importance of differentiating anger proneness from habitual styles of anger expression when examining the link between anger and psychosomatic complaints. Anger proneness is considered to be a relatively stable personality trait and is defined as a tendency to experience angry feelings (Spielberger et al., 1985). Individuals with high levels of anger proneness tend to perceive a wider range of situations as anger-eliciting situations and to experience more persistent anger during these situations than individuals with low levels of anger proneness. Comparatively, anger expression refers to people’s habitual modes of expressing angry feelings. Spielberger et al. (1985) proposed two basic dimensions of anger expression, anger-in and anger-out. Anger-in refers to the extent to which people ruminate over and suppress angry feelings rather than expressing them overtly. By contrast, anger-out refers to the extent to which people openly express their anger to other people or the environment.

Prior research has empirically established the paths from anger proneness and inward anger expression styles to the development and maintenance of OC symptoms. Whiteside and Abramowitz (2004) investigated 131 undergraduates and found that individuals high in OC checking symptoms tended to experience more anger and were more likely to suppress anger. Furthermore, their subsequent study indicated that patients with OCD displayed higher levels of anger than healthy controls (Whiteside and Abramowitz, 2005). A study of 33 patients with OCD and 143 college students demonstrated that OCD patients with compulsive checking symptoms reported greater trait anger than the student control group, and OC symptoms were positively correlated with the suppression of angry feelings (Radomsky et al., 2007). Tellawi et al.’s (2016) research revealed that individuals with OCD had higher levels of hostility and anger, which in turn were significantly positively associated with increased OCD severity. Similarly, Piacentino et al. (2016) found an association between anger and specific obsession subtypes in OCD patients. Some of these previous studies belong to cross-sectional investigations and could not determine causality. Nevertheless, the fact that anger proneness and expression styles are relatively stable personality traits and OC symptoms are more regarded as temporal mental health distress partially supports the validity of the pathway from anger to OC symptoms (Spielberger et al., 1985).

### Family Cohesion, Family Adaptability, and OC Thoughts and Behaviors

Studies have also partially supported empirical links between family environment-related variables, such as family cohesion, and OCD. Based on their family therapy practice experience and research on slum families, Minuchin et al. (1978) defined the dynamics of psychosomatic families as boundary confusion, rigid behavioral control, and poor adaptability. Gorenstein et al. (2015) assessed the impact of child-focused pediatric OCD treatment on the family environments of 33 children and found that family cohesion was correlated with the children’s clinical improvement. A review on the relationships between the broader class of obsessive compulsive and related disorders (OCRDs) and family environment suggested that pediatric clients with OCRDs had family environments with low levels of cohesion and experienced more domestic violence within the family (Murphy and Flessner, 2015). Peris and Piacentini (2013) conducted positive family interaction therapy with 20 youth diagnosed with OCD and found a reliable reduction in symptoms as family function improved. A study examining the outcomes of family-focused cognitive behavioral therapy for adolescents with OCD demonstrated that higher family cohesion predicted positive responses to psychotherapy (Pens et al., 2012).

Comparatively, the empirical support for association between family adaptability and OC thoughts and behaviors is limited. Jimenez et al. (2011) assessed 48 OCD patients and found that the patients and their relatives’ comprehension of the disorder increased in proportion to a higher family adaptation. The results implied that higher family flexibility might be related with less OC complains. However, causal relationship between family adaptability and OC symptoms has not been empirically established in prior research. These results imply that family adaptability might be indirectly related to OC symptoms.

### Family Cohesion and Adaptability, Anger Proneness, and Anger Expression

Prior research has not established adequate and consistent empirical associations between family cohesion, adaptability and anger trait and expression styles. Houltberg et al. (2012) investigated 84 normal children and adolescents, and their study yielded no direct association between family cohesion or adaptability and anger regulation. A study examining the influence of original family environment upon young adulthood’s response to interpersonal conflict found that participants rating extremely low in family adaptability and cohesion exhibited greater anger. However, the study only explored the association between family circumstance and current anger experience without examining the links between anger trait and expression and family variables (Larkin et al., 2011).

### The Effects of Family Function on the Relationship between Trait Anger and Anger Suppression and OCD

A number of studies and theories have assessed whether OC symptoms are associated with anger proneness, habitual styles of anger expression and family environment. Moreover, as described in McGoldrick et al.’s (2008) transgenerational theory, college students are in the first transference stage of the family life cycle. A core developmental task that young adults face during this stage is differentiating their own affective experience and cognitive-behavioral styles from those of their family of origin. Such differentiation results in gradually increased connections between their psychosomatic and mental health symptoms and individual personality characteristics. Meanwhile, young adults remain affectively or cognitively connected to their families. Hence, the contribution of anger expression and proneness, which are considered relatively stable personality traits, to the development of OC symptoms might be comparable to that of family environment-related variables. However, the mechanism underlying how the interaction between personality traits and family climate variables impacts the relationship between anger and OC symptoms remains unclear.

According to Baron and Kenny’s (1986) theory, the requisite conditions for testing the mediational hypotheses should be that independent variable (X), the mediator (Z) and the dependent variable(Y) should strongly correlate with each other. Although the associations between family cohesion and OCD, and between OC symptoms and both anger proneness and anger suppression were demonstrated, the empirical support for links between family environment variables and anger trait and regulation is inadequate. It implies that the requisite conditions for testing whether anger suppression or anger proneness would mediate the link between family circumstance and OC symptoms could not be met. Neither is testing the mediational roles of family cohesion and family adaptability in the associations between anger trait and expression and OC symptoms warranted.

Comparatively, as for the conditions of establishing moderation model (Baron and Kenny, 1986), the independent variable (X) should be causally antecedent to the dependent variable (Y). Additionally, it is desirable that the moderator variable (Z) should be uncorrelated with both the independent variable (X) and the dependent variable(Y). As discussed in the theory of family therapy and previous research, family function and family dynamics contribute to both the etiology and maintenance of mental health pathologies (Minuchin et al., 1978; Retzlaff et al., 2013). Meanwhile, prior research has established empirical links between anger and OC symptoms, and the empirical evidence for associations between family variables and anger trait and regulation is inadequate. It implies that the preconditions for testing whether family variables would moderate the link between anger trait and expression and OC symptoms could be hold. Thus, the present study examines family cohesion and family adaptability as moderators of the relationship between anger proneness and expression and reports of OC symptoms in a sample of college-based students. We attempted to test whether the variances in family cohesion and family adaptability affect the strength of the relationship between anger and OC symptoms. We hypothesized that higher levels of family cohesion or flexibility might attenuate the associations between anger proneness and its habitual expression and OC symptoms. Furthermore, anger experience and trait anger might be more significantly correlated with OC symptoms among individuals with lower levels of family cohesion or adaptability.

In light of empirical evidence indicating that the links between anger experience; style of anger expression; and psychosomatic symptoms, such as somatic complains, differ between men and women (Liu et al., 2011), we conducted the analysis independently for men and women. However, due to the lack of empirical research investigating gender differences in the association between anger and OCD or OC symptoms, our research takes an exploratory analysis approach without positing a concrete hypothesis.

Additionally, we controlled for several factors that have been linked to both OCD and other psychosomatic symptoms: age, socioeconomic status, ethnicity, and one-child family status (Murphy and Flessner, 2015; Fan, 2016). Finally, we also examined current levels of depression and anxiety both because these two factors are commonly associated with OC symptoms and anger proneness and suppression (Radomsky et al., 2007; Breen and Kashdan, 2011; Yap et al., 2012; Nissen et al., 2016) and because depressive and anxious symptoms may bias participants toward more negative responses to other assessments, including inventories of OCD and family circumstance (Waldinger et al., 2006).

In contrast to the majority of prior studies, which examined only clinical samples, the present study focuses on a sample of college students. Compared to clinical samples, which are likely to have higher levels of medical illness, a college-based sample offers a greater opportunity to examine the links between anger and OC symptoms across a spectrum of normal health status. Considering our sample of normal college students, we chose a dimensional rather than categorical method of assessing the subjects’ mental health status because continuous measures provide a greater opportunity to investigate vivid connections between variables.

Finally, because this study was conducted as part of the comprehensive survey on the students’ mental health status at the beginning of the new semester, we just used SCL-90 to assess the participants’ OC, depressive and anxious symptoms. Although SCL-90 has been widely used in Chinese college students (Huang and Li, 2009), it is still issued more as a general measure of the emotional distress. Thus, it might not be the best measure of OC symptoms because some of this dimension may even be frequently presented in the general population. So, we must be circumspect when explaining our results.

## Materials and Methods

### Participants and Procedures

A total of 2600 college students in one comprehensive university of Shanghai were invited to participate in the study from Feb to May 2016, during the beginning of the new semester. As demonstrated by prior study, Chinese students in this period of the term reported less academic stress. So, it guaranteed a better controlled sample without the influence of other cofounding variables such as exam stress. 46 students refused participation and 18 subjects were excluded due to physical disease (diagnosed by at least 2 physicians) or mental health disorders (screened by at least 2 psychiatrists according to ICD-10), with reference to their physical exam database established at the beginning of the college semester. Thus, 2536 students were enrolled in the study. Each participant provided written informed consent at the beginning of the study. Participants then completed questionnaires that included measurements of their demographic data, trait anger and anger expression models, family cohesion, family adaptability and symptoms, including obsession, depression and anxiety. The effective response sample was 2008 participants (1128 male and 880 female) which accounted for effective rate of 79.2%. This study was approved by the institutional review board of Tongji University’s School of Medicine.

The participants’ mean age was 19.8 years (*SD* = 0.9). 89.6% of the participants were of Han nationality, and 10.4% were of minority nationality. Of the participants, 52.6% were freshmen and 47.4% were sophomore. 1477 (73.6%) students came from one-child families.

### Measures

#### Demographics

Age, gender, grade, major, family income, ethnicity, education level of parents, one-child family status data were obtained through written questionnaires.

#### Obsessive and Compulsive Symptoms

Obsessive and compulsive symptoms and severity were measured using the Symptom Check List-90 (SCL-90). This self-report scale includes 90 items rated on a 5-point Likert scale that assess the degree to which the individual is distressed by different symptoms over the prior one week (1 = not at all, 5 = extremely). The items are classified into nine primary symptom dimensions, including OC symptoms, depression and anxiety. Continuous scores on the OC symptoms, depression and anxiety subscales were derived by computing the mean rating of the items for each scale. A higher score indicates more severe psychological symptoms. The adequate test–retest reliability, internal consistency reliability, and convergent, content and external validity of the SCL-90 have been established in Chinese college students (Huang and Li, 2009; Shi et al., 2013). In Shi et al.’s (2013) study on 8265 Chinese college students, the internal consistency reliabilities (α coefficients) of OC, depression, and anxiety subscales were 0.82, 0.87, and 0.83. Additionally, the scores of OC, depression and anxiety subscales have been associated with the life stress, dysfunctional personality traits and the diagnosis of OCD in Chinese population (Luo et al., 2000; Li and Chen, 2003; Liu, 2008). However, considering the fact that SCL-90 has been mainly used as a general measure of emotional distress, it might not be the best measure of OC symptoms and has no diagnostic properties. We must be circumspect about the generalizability of our findings to the general population, such as the clinical sample.

#### Anger Proneness and Anger Expression

Trait anger was assessed using the State-Trait Anger Expression Inventory 2 (STAXI-2, Chinese version). This self-report scale was developed by Spielberger (1999) and translated into Chinese by Liu and Gao (2012). The STAXI-2 contains 57 items that are rated on a 4-point Likert scale. The items are classified into nine dimensions that measure an individual’s state anger, trait anger and anger expression styles. In line with our research aims, the continuous scores derived from the *Anger-in* and *Anger-out* subscales were used to index anger expression styles. *Anger-in* refers to the extent to which people mentally stew over angry feelings without expressing them overtly and is an index of the degree to which individuals tend to suppress anger. By contrast, *anger-out* concerns the extent to which people express their anger openly. We used the scores of the *Trait anger*/*trait* and *Trait anger/response* subscales to demonstrate participants’ anger proneness. *Trait anger*/*trait* refers to one’s proneness to generally experience angry feelings without specific stress or stimulation. *Trait anger/response* concerns an individual’s proneness to experience angry feelings when confronted with frustrating situations and criticism. Liu and Gao’s study demonstrated the good internal consistency reliability, test–retest reliability, convergent validity, and external validity of the STAXI-2 subscales with the exception of the internal consistency reliabilities of *anger-in* and *anger-out* (Liu and Gao, 2012). In the current study, the internal consistency reliabilities (α coefficients) of *anger-in* and *anger-out* were 0.84 and 0.72, respectively.

#### Family Cohesion and Family Adaptability

The Family Adaptability and Cohesion Scale, second edition (FACES II, Chinese Version) was used to measure the cohesion and adaptability of the participants’ family of origin. The FACES-II was imported into China by Phillips et al. (1991). It is a self-report scale composed of 30 items Participants rated how well each of the items described their families of origin on a 5-point Likert-type scale ranging from never (1) to always (5). Continuous scores on the two factor-analytically derived subscales indexing *Family cohesion* and *Family adaptability* were used. *Family cohesion* refers to the extent to which family members remain emotionally close to each other. *Family adaptability* concerns families’ ability to adapt to different challenges and situations resourcefully and flexibly. Higher scores are positively related to higher levels of trait family closeness and family reflexivity, which refers to problem solving. Phillips et al. (1991, 1999) surveyed 325 Chinese paticipants which included 102 schezophrenia patients and relatives, and 223 health control subjects using this scale. They found that FACES-II (Chinese version) demonstrated good discriminant and convergent validity, and the internal consistency reliabilities (α coefficient) of *Family cohesion* and *Family adaptability* subscale were 0.85 and 0.73, respectively. The two subscales have also shown adequate test–retest reliability (between 0.84 and 0.91).

### Statistical Analysis

SPSS 19.0 was used to conduct the statistical analysis. Correlational and regression analysis were conducted separately for male and female participants. One-way ANOVA was used to compare the scores of OC symptoms, depression, anxiety, ager trait, anger expression, family cohesion and family adaptability between genders. Links between anger trait, anger expression, depression, anxiety, family cohesion and family adaptability, and OC symptoms were assessed with Pearson correlation coefficients. Then, we conducted multivariate linear regression analysis (Enter) to identify which of the factors, *trait anger/trait*, *trait anger/response*, *anger-in* and *anger-out*, significantly predict OC symptoms when their confounding influence on each other is controlled. Finally, based on the theory of Baron and Kenny (1986), the significances of potential moderators, family cohesion and family adaptability, were identified using multivariate linear regression analysis (Enter). If the independent variable is denoted as X, the moderator as Z, and the dependent variable as Y, Y is regressed on X, Z, and X^∗^Z. Moderator effects are indicated by the significant effect of X^∗^Z while X and Z are controlled. All statistical tests were two-tailed and the level of statistical significance was set at *p* < 0.05.

## Results

In our sample, the mean OC scores were 1.71 (*SD* = 0.71) for male students and 1.67 (*SD* = 0.64) for female students. One-way ANOVA revealed that female students reported higher *family cohesion* (*F* = 35.76, *df* = 1903, *p* < 0.001), *family adaptability* (*F* = 11.84, *df* = 1928, *p* = 0.001), *trait anger*/*trait* (*F* = 6.59, *df* = 1984, *p* = 0.01) and anger-in (*F* = 5.89, *df* = 1939, *p* = 0.015) scores and lower *anger-out* (*F* = 4.62, *df* = 1961, *p* = 0.032) scores than their male counterparts. No significant differences in current depression, anxiety, OC symptoms or *trait anger*/*response* were found between males and females.

### Correlations between Variables in the Moderation Model

Pearson correlations revealed that OC symptom scores were positively associated with *trait anger/trait, trait anger/response, anger-in, anger-out*, depression and anxiety for both male and female students (see **Table 1**). The correlation coefficients (*r*) of the connections between *trait anger/trait, trait anger/response, anger-in* and OC symptoms ranged from 0.39 to 0.50 (*P*s < 0.001). For both men and women, OC symptoms were negatively associated with *family cohesion* and *family adaptability, with* the correlation coefficients (*r*) ranging from –0.28 to –0.16. Meanwhile, for both genders, *trait anger/trait, trait anger/response, anger-in* and *anger-out* were negatively related with *family cohesion* and *family adaptability (r* = –0.13 to –0.06, *Ps* < 0.001*).* The coefficients of the correlations between family environment variables and anger proneness/expression and OC symptoms were relatively small, despite statistically significant. These results implied that the requisite conditions identified by Baron and Kenny (1986) were met for testing whether *family cohesion* and *adaptability* would moderate the links between anger and OC symptoms. However, testing the meditational roles of *family cohesion* or *family adaptability* in the links between anger and OCD was not warranted.

**Table 1:** Pearson correlations between obsessive–compulsive (OC) symptoms and variables in the moderation model.

	OC symptoms			
	
	Male (*n* = 1128)		Female (*n* = 880)	
		
	*r*	*p*	*r*	*p*
Age	-0.02	0.48	0.00	0.99
Family income	-0.04	0.22	-0.06	0.09
Ethnicity	0.04	0.24	-0.01	0.70
One-child family	0.07	0.014	0.10	0.003
Trait anger/trait	0.45	<0.001	0.39	<0.001
Trait anger/response	0.50	<0.001	0.41	<0.001
Anger-in	0.43	<0.001	0.41	<0.001
Anger-out	0.26	<0.001	0.19	<0.001
Depression	0.77	<0.001	0.75	<0.001
Anxiety	0.76	<0.001	0.74	<0.001
Family cohesion	-0.16	<0.001	-0.25	<0.001
Family adaptability	-0.19	<0.001	-0.28	<0.001


Links between contextual variables and OC symptoms were also examined. As shown in **Table 1**, one-child family status was significantly correlated with SCL-90 OC subscale scores for both male and female students, whereas associations between OC symptoms and age, family income and ethnicity were nonsignificant. These potential confounding variables were included as covariates in subsequent analyses to control for their influence.

### Testing the Independent Predictors of OC Symptoms

At the second step of our analysis, multivariate linear regression analysis (Enter) was conducted to identify which of the factors, *trait anger/trait*, *trait anger/response*, *anger-in*, and *anger-out*, significantly predict OC symptoms when their confounding influence on each other is controlled. The analysis showed that for the male subjects, both *trait anger/response* (β = 0.17, *p* < 0.001) and *anger-in* (β = 0.06, *p* = 0.004) were significantly linked with OC scores after controlling for the other anger-related variables in the model. For female students, only *anger-in* remained a significant predictor of OC symptoms after controlling for the other anger variables (β = 0.10, *p* < 0.001).

### Testing the Moderating Effects of Family Cohesion and Family Adaptability in the Link between Trait Anger and OC Symptoms for Males

Based on the guidelines established by Baron and Kenny (1986) and elaborated by Kraemer et al. (2001), we tested the potential moderating effects of *family cohesion* and *family adaptability* separately for the male and female students. If the independent variable is denoted as X (*trait anger* or *anger-in*), the moderator as Z (*family cohesion* or *family adaptability*), and the dependent variable as Y (OC symptoms), Y is regressed on X, Z, and X^∗^Z. Moderator effects are indicated by the significant effect of X^∗^Z while X and Z are controlled. Follow-up *simple slope analysis* was carried out to clarify the directions of the moderation effect (Aiken and West, 1991).

**Table 2** presents the results of multivariate linear regressions that tested whether *family cohesion* or *family adaptability* moderated the links between *trait anger/response* and OC symptoms for male students. Age, family income, ethnicity, one-child family status, current depression and anxiety were introduced as covariates. The results demonstrated that for male students, the standardized regression coefficient (β) of *family cohesion^∗^trait anger/response* remained significant while *trait anger/response* and *family cohesion* were controlled, indicating that *family cohesion* moderated the link between male subjects’ trait anger and OC symptoms. By contrast, the β of *family adaptability^∗^trait anger/response* was statistically nonsignificant, demonstrating that *family adaptability* did not moderate the association between *trait anger/response* and OC symptoms.

**Table 2:** Moderating effects of family cohesion and family adaptability in the link between Trait anger/response and OC symptoms among males.

	OC symptoms						

	Family cohesion				Family adaptability		
			
	β	*t*-value	*R*^2^		β	*t*-value	*R*^2^
			0.67				0.66
Age	0.01	0.48		Age	0.00	0.20	
One child	0.00	0.19		One child	-0.00	-0.06	
Ethnicity	0.00	0.02		Ethnicity	0.00	0.10	
FI	-0.03	-1.59		FI	-0.04	-1.66	
Depression	0.41	12.04***		Depression	0.39	11.25***	
Anxiety	0.34	10.14***		Anxiety	0.36	10.49***	
TA	0.16	1.13		TA	0.06	0.54	
FC	-0.11	-1.97*		FA	-0.04	-0.63	
TA ^∗^ FC	-0.36	-2.54**		TA ^∗^ FA	-0.14	-1.15	


*FI, Family income; FC, Family cohesion; FA, Family adaptability; TA, Trait anger/response. ^∗∗∗^*p* < 0.001, ^∗∗^*p* < 0.01, ^∗^*p* < 0.05. βs reported here are standardized regression coefficients.*

The direction of the moderating effect of *family cohesion* in the relationship between anger and OC symptoms was further explored using *simple slope analyses*. The male students were conditioned at 1 standard deviation above and below the mean on *family cohesion* scores. When conditioned at one standard deviation below the mean on *family cohesion* and when the influence of the above-mentioned confounding variables were controlled, higher *trait anger/response* was significantly related to more OC behaviors and thoughts, β = 0.17, *t*(126) = 2.73, *p* = 0.007. When conditioned at one standard deviation above the mean on *family cohesion*, high *trait anger/response* had a nonsignificant relationship with OC symptoms, β = 0.12, *t*(124) = 1.91, *p* = 0.06.

### Testing the Moderating Effects of Family Cohesion and Family Adaptability in the Link between Anger-in and OC Symptoms

**Table 3** shows the results of similar models testing *family adaptability* as a potential moderator of the link between *Anger-in* and OC symptoms for males and females. For female students, the significant β of *anger-in^∗^family adaptability* suggested that *family adaptability* moderated the link between *anger-in* and OC thoughts and behaviors. Similarly, the moderating effect was further clarified using *simple slope analyses* and conditioned at 1 SD above and below the mean on *family adaptability* scores. When conditioned at one standard deviation below the mean on *family adaptability*, higher *anger-in* was significantly linked to higher OC scores, β = 0.14, *t*(112) = 1.98, *p* = 0.04. When conditioned at one standard deviation above the mean on *family adaptability*, high *anger-in* had a nonsignificant association with OC symptoms, β = 0.08, *t*(139) = 1.30, *p* = 0.20.

**Table 3:** Moderating effects of family adaptability in the association between Anger-in and OC symptoms.

	OC symptoms					

	Male (*n* = 1128)			Female (*n* = 880)		
			
	β	*t*-value	*R*^2^	β	*t*-value	*R*^2^
			0.64			0.62
Age	-0.00	-0.15		0.00	0.06	
One child	0.00	0.30		0.02	0.69	
Ethnicity	0.01	0.40		-0.03	-1.30	
FI	-0.04	-1.61		0.01	0.27	
Depression	0.39	10.78***		0.40	9.78***	
Anxiety	0.41	11.71***		0.38	9.92***	
AI	0.04	0.33		0.18	1.37	
FA	-0.04	-0.63		-0.15	-1.96	
AI ^∗^ FA	-0.14	-1.10		-0.30	-2.24*	


*FI, Family income; FA, Family adaptability; AI, Anger-in. ^∗∗∗^*p* < 0.001, ^∗∗^*p* < 0.01, ^∗^*p* < 0.05. βs reported here are standardized regression coefficients.*

The moderating effect of *family adaptability* in the same association was nonsignificant for the male subjects, β = 0.14, *t*(991) = 1.10, *p* = 0.27 (see **Table 3**).

As shown in **Table 4**, the βs of *anger-in^∗^family cohesion* were nonsignificant for both male and female students after controlling for the influence of current depression and anxiety. This result demonstrated that the link between *anger-in* and OC symptoms was not moderated by *family cohesion* in the present sample.

**Table 4:** Moderating effects of family cohesion in the association between Anger-in and OC symptoms.

	OC symptoms					

	Male (*n* = 1128)			Female (*n* = 880)		
			
	β	*t*-value	*R*^2^	β	*t*-value	*R*^2^
			0.65			0.61
Age	0.00	0.18		0.00	-0.01	
One child	0.01	0.70		0.02	0.72	
Ethnicity	0.01	0.41		-0.03	-1.42	
FI	-0.03	-1.64		-0.03	-0.88	
Depression	0.41	11.50***		0.40	9.79***	
Anxiety	0.40	11.35***		0.38	9.87***	
AI	-0.17	-1.17		-0.19	-1.20	
FC	-0.09	-1.24		-0.10	-1.23	
AI ^∗^ FC	0.27	1.82		0.31	1.91	


*FI, Family income; FC, Family cohesion; AI, Anger-in. ^∗∗∗^*p* < 0.001, ^∗∗^*p* < 0.01, ^∗^*p* < 0.05. βs reported here are standardized regression coefficients.*

## Discussion

Our initial analyses replicated the independent associations between adult OC symptoms and anger proneness, anger expression styles, depression, anxiety, *family cohesion* and *family adaptability* found in prior research (Whiteside and Abramowitz, 2004, 2005; Pens et al., 2012; Yap et al., 2012; Peris and Piacentini, 2013; Gorenstein et al., 2015; Murphy and Flessner, 2015; Nissen et al., 2016; Piacentino et al., 2016; Tellawi et al., 2016).

When including all trait anger subtypes and anger expression styles in the same regression model, we found that *trait anger/response* and *anger-in* were significantly related to OC symptoms among male subjects. By contrast, for women, only *anger-in* significantly predicted OC thoughts and behaviors. The independent contribution of *anger-in* found in both genders is consistent with the results of previous research (Whiteside and Abramowitz, 2004, 2005). This finding implied that the stifling of angry feelings may prompt a compensatory focus on inner bodily sensations, leading individuals to develop OC rituals or thoughts to release their suppressed anger (Whiteside and Abramowitz, 2005). This explanation is also somewhat consistent with Gross and Levenson’s (Gross and Levenson, 1997) studies on the physiological effects of the suppression of negative emotions. Additionally, the special predictive effect of *trait anger/response* on the development of men’s OC symptoms found in the current study supports the conclusions of prior study (Whiteside and Abramowitz, 2004, 2005; Tellawi et al., 2016), suggesting that more frequent and prolonged experiences of anger may lead to higher levels of OC symptoms in men. However, further research is warranted to clarify why proneness to anger may be a particularly salient predictor of men’s OC behaviors and thoughts. Socialized gender stereotypes, which posit that men are more likely to experience anger, do not appear to explain our results, as evidenced by the absence of significant gender differences in trait anger scores (Jiang et al., 2010; Yao et al., 2011).

This study is the first to address the moderating effects of family cohesion and family adaptability in the link between anger proneness and anger expression and OC symptoms. Our main findings were as follows: in a college-based sample, family cohesion moderated the link between trait anger and OC symptoms among the male students, whereas family adaptability moderated the link between anger-in and OC among female subjects. How can we explain these moderation effects? In Chinese culture, the socialized gender stereotype of men posits that they are more comfortable expressing their anger outwardly (Jiang et al., 2010; Yao et al., 2011). When enmeshed in an environment where anger is overtly displayed, it might be more natural and acceptable for men to openly express their anger in the family environment. In a family atmosphere with a high level of interpersonal support, men might be able to receive more empathic responses or support from previous caregivers and families. Hence, this greater family cohesion could provide men with more resources and help to protect them against the attack of anger-eliciting experiences. This explanation is somewhat supported by our study’s finding that the male students reported higher *anger-out* scores than the female subjects. It is also consistent with previous research on the outcomes of resource-oriented family therapy approaches, which found that OCD patients benefit from psychotherapy models that explore and cultivate the family’s resources, e.g., by fostering increased family cohesion (Pens et al., 2012; Peris and Piacentini, 2013).

With respect to the female subjects, the traditional Chinese culture might view them as more obedient and modest (Jiang et al., 2010; Yao et al., 2011). The view that the overt expression of anger makes one less lovable and impolite and might drive away or anger one’s friends or families may prompt female students to suppress angry feelings to maintain a good appearance and ties to needed others. Thus, women embedded in a family environment with greater flexibility in coping with daily challenges and emotional stress might learn more strategies to manage their own angry feelings beyond suppressing anger, reducing the risk of developing OC symptoms. Meanwhile, previous research implied that perfectionism and intolerance of uncertainty beliefs were associated with women’s OCD symptoms (Radomsky et al., 2007). Thus, higher levels of family adaptability could increase females’ resilience and skills for coping with negative feelings and reduce their anxiety regarding imperfections and uncertainty. This might also reduce females’ risk of developing OC symptoms.

Due to the correlational links between depression, anxiety and OC symptoms (Yap et al., 2012; Nissen et al., 2016), the inclusion of depressive and anxious symptoms in the moderation regressions provided a particularly stringent test of our models. Of note, even after accounting for current depression and anxiety, the moderating effects of anger suppression and anger proneness remained significant. This result is to some degree inconsistent with Whiteside and Abramowitz’s (2004) conclusions that among patients with high levels of OC symptoms, the relationship between anger experience and OCD symptoms was mainly attenuated by comorbid depressive emotions. This inconsistency may be due to differences in the samples. The individuals diagnosed with OCD might suffer from more severe depression or anxious feelings, potentially amplifying the influence of these negative emotions. Hence, the predictive effect of angry feelings became less significant after accounting for current depression.

### Limitations

Our study has several limitations. First, we explored only the link between general OCD symptoms and anger. Whiteside and Abramowitz (2004, 2005) underscored the importance of distinguishing the associations between anger and different OC symptoms, including checking, doubting, washing and slowness. Thus, further research that considers the diversity of OCD symptoms is strongly recommended (Whiteside and Abramowitz, 2004, 2005; Piacentino et al., 2016). Second, in the present study, we selected college students, who might be higher functioning, better educated and generally healthier than a more mixed population including individuals with mental health disorders or interpersonal difficulties. These students might also display a higher level of mentalization than the general population. Thus, we must be circumspect about the generalizability of our findings to the general population. Future studies should explore the same moderation model in a more mixed and general sample. Furthermore, future studies are needed to replicate our findings in a clinical OCD population. Third, although the SCL-90 has been frequently used in college student samples and our study suggested that the moderating effects of family cohesion and family adaptability remained significant after controlling for the influence of current depression and anxiety, the SCL-90 does not differentiate between OC, depressive symptoms and anxious symptoms very well. Some of the items in the OC dimension may even be frequently present in the general population. Thus, further research should utilize measures with greater discriminative validity to ensure OC symptom specificity. Fourth, we did not control for other potential covariates that might have an influence on the development and maintenance of OC symptoms. For example, previous research has revealed that domestic violence within the family, cognitive schema, parental symptoms of anxiety and depression, family strain and stress influence current level of OCD symptoms (Whiteside and Abramowitz, 2005; Radomsky et al., 2007; Murphy and Flessner, 2015). Future studies incorporating measures of more potential covariates are recommended. Fifth, in the current study, all the psychosomatic variables were assessed by self-report scales. Self-report scale scores might be subjective. Thus, some other types of evaluation scales should be used in future research to increase objectivity. Sixth, the cross-sectional design of the study establishes associations but cannot determine causality. Although the path from anger to OC symptoms and the moderating effects of family cohesion and family adaptability make sense temporally, other explanations or paths are also possible. For example, OC symptoms may cause subjects to become more irritated, which in turn might contribute to the development of OC symptoms. Moreover, it might also be possible that family circumstance leads to high anger experience and suppression and, in turn, foster more OC symptoms. Prospective studies are needed to shed light on the causal relationships between anger, family function and OC symptoms.

### Clinical Implications

Research revealing mechanisms underlying the link between anger experience and expression styles and OCD may inform the psychiatric treatment of individuals with trait anger who report OC symptoms. Because anger proneness and anger-in are relatively stable personality traits and may be difficult to change (Spielberger et al., 1985), it might be more productive for therapists to focus on potentially modifiable factors by fostering, e.g., more empathetic and supportive family emotional climates for male clients. Peris and Piacentini’s (2013) reported that OCD patients benefitted from psychological interventions that cultivated more positive and flexible family interaction. Similarly, the results of current study suggest that female OCD patients who are accustomed to suppressing their anger might benefit from interventions that help their family to develop more flexible and resilient strategies for coping with negative feelings, potentially reducing their vulnerability to OC symptoms.

## Author Contributions

LL contributed to the program design, subject screening, data inputting and analysis, article writing and editing. CL distributed the measurements and inputted the data. XZ has contributed to the program design, screening of subjects and editing of articles.

## Conflict of Interest Statement

The authors declare that the research was conducted in the absence of any commercial or financial relationships that could be construed as a potential conflict of interest.
